# A National Registry of Thalassemia in Turkey: Demographic and Disease Characteristics of Patients, Achievements, and Challenges in Prevention

**DOI:** 10.4274/tjh.2017.0039

**Published:** 2018-03-06

**Authors:** Yeşim Aydınok, Yeşim Oymak, Berna Atabay, Gönül Aydoğan, Akif Yeşilipek, Selma Ünal, Yurdanur Kılınç, Banu Oflaz, Mehmet Akın, Canan Vergin, Melike Sezgin Evim, Ümran Çalışkan, Şule Ünal, Ali Bay, Elif Kazancı, Talia İleri, Didem Atay, Türkan Patıroğlu, Selda Kahraman, Murat Söker, Mediha Akcan, Aydan Akdeniz, Mustafa Büyükavcı, Güçhan Alanoğlu, Özcan Bör, Nur Soyer, Nihal Özdemir Karadaş, Ezgi Uysalol, Meral Türker, Arzu Akçay, Süheyla Ocak, Adalet Meral Güneş, Hüseyin Tokgöz, Elif Ünal, Naci Tiftik, Zeynep Karakaş

**Affiliations:** 1 Hemoglobinopathy Study Group, Turkey

**Keywords:** Thalassemia, Hemoglobinopathies, Splenectomy, Registries, Iron chelators, β-thalassemia mutations, Turkey

## Abstract

**Objective:**

The Turkish Society of Pediatric Hematology set up a National Hemoglobinopathy Registry to demonstrate the demographic and disease characteristics of patients and assess the efficacy of a hemoglobinopathy control program (HCP) over 10 years in Turkey.

**Materials and Methods:**

A total of 2046 patients from 27 thalassemia centers were registered, of which 1988 were eligible for analysis. This cohort mainly comprised patients with β-thalassemia major (n=1658, 83.4%) and intermedia (n=215, 10.8%).

**Results:**

The majority of patients were from the coastal areas of Turkey. The high number of patients in Southeastern Anatolia was due to that area having the highest rates of consanguineous marriage and fertility. The most common 11 mutations represented 90% of all β-thalassemia alleles and 47% of those were IVS1-110(G->A) mutations. The probability of undergoing splenectomy within the first 10 years of life was 20%, a rate unchanged since the 1980s. Iron chelators were administered as monotherapy regimens in 95% of patients and deferasirox was prescribed in 81.3% of those cases. Deferasirox administration was the highest (93.6%) in patients aged <10 years. Of the thalassemia major patients, 5.8% had match-related hemopoietic stem cell transplantation with a success rate of 77%. Cardiac disease was detected as a major cause of death and did not show a decreasing trend in 5-year cohorts since 1999.

**Conclusion:**

While the HCP has been implemented since 2003, the affected births have shown a consistent decrease only after 2009, being at lowest 34 cases per year. This program failure resulted from a lack of premarital screening in the majority of cases. Additional problems were unawareness of the risk and misinformation of the at-risk couples. In addition, prenatal diagnosis was either not offered to or was not accepted by the at-risk families. This study indicated that a continuous effort is needed for optimizing the management of thalassemia and the development of strategies is essential for further achievements in the HCP in Turkey.

## Abstract (tr)

**Amaç:**

Türk Pediatrik Hematoloji Derneği, Türkiye’de 10 yıldır devam eden Hemoglobinopati Kontrol Programı’nın (HCP) etkinliğini değerlendirmek ve hemoglobinopati hastalarının demografik ve hastalık özelliklerini ortaya koymak üzere bir Ulusal Hemoglobinopati Kayıt Programı oluşturdu.

**Gereç ve Yöntemler:**

Toplam 27 talasemi merkezinden 2046 hasta kaydedildi ve bunların 1988’i analize uygun bulundu. Kayıtların çoğunluğunu β-talasemi majör (n=1658, %83,4) ve intermedia (n=215, %10,8) olguları oluşturdu.

**Bulgular:**

Hastaların büyük çoğunluğu kıyı bölgelerde bulunuyordu. Güneydoğu Anadolu’da yüksek hasta sayısına, bu bölgede en yüksek görülen akraba evliliği ve yüksek doğum oranının etkisi olabilirdi. En sık 11 talasemi mutasyonu, tüm β-talasemi allellerinin %90’ını oluşturuyordu ve bunların %47’si IVS1-110(G->A) mutasyonu idi. Yaşamın ilk 10 yılında splenektomi olasılığı %20 idi ve bu oran 1980’lerden beri değişmemişti. Demir şelasyonu hastaların %95’inde monoterapi olarak uygulanmaktaydı ve %81,3’ünü deferasiroks oluşturuyordu. Deferasiroks uygulaması en yüksek (%93,6) 10 yaştan küçük hastalarda bulundu. Talasemi majör olgularının %5,8’i hemopoetik kök hücre nakli olmuştu ve başarı oranı %77 idi. Kardiyak hastalık ölümlerin majör nedeni idi ve beşer yıllık kohortlarda, 1999’dan beri azalma eğilimi göstermiyordu.

**Sonuç:**

HCP 2003 yılından beri uygulanmakla beraber, yeni hastaların doğumu ancak 2009’dan itibaren azalma eğilimi gösteriyordu ve en düşük yılda 34 yeni hasta saptandı. Program başarısızlığı, çiftlerin çoğunda, evlilik öncesi tarama yapılmamasından kaynaklanmaktaydı. Bir kısmında ise riskin farkında olunmaması ve çiftlerin hatalı bilgilendirilmesi nedenliydi. Sonuç olarak, risk ailelerine prenatal tanı önerilmemesi veya prenatal tanının reddi diğer nedenleri oluşturuyordu. Bu çalışma, Türkiye’de talasemi tedavisinin optimizasyonu için çabanın sürdürülmesine ve HCP’nin daha yüksek başarısı için gelişen stratejilere gereksinim olduğunu gösterdi.

## Introduction

Better management of thalassemia by regular and adequate red cell transfusions, close monitoring of iron loading, and appropriate iron chelation therapy (ICT) with deferoxamine (DFO) has changed the prognosis of the disease worldwide [1]. Furthermore, there was a revolutionary development in the management of the disease at the beginning of the twenty-first century with the introduction of magnetic resonance imaging (MRI) as a measure of tissue-specific iron loading and the availability of oral iron chelators deferiprone (DFP) and deferasirox (DFX) [2,3]. 

In parallel, DFP and DFX were registered in Turkey in 2004 and 2006, respectively, and gradually replaced DFO. However, the dissemination of cardiac T2* MRI as a useful tool for the monitoring and management of iron overload has remained limited.

The cornerstone of relevant public health policies in Turkey was the recognition of thalassemia as a common health problem in 1993. Eventually, a comprehensive national hemoglobinopathy control program (HCP) was implemented by law and came into force on 24 October 2002 in 33 provinces of Turkey. 

In 2012, the Turkish Society of Pediatric Hematology set up the National Registry for Hemoglobinopathies to collate the demographic and disease characteristics of patients, and also quantified and assessed the efficacy of the HCP over 10 years in Turkey.

## Materials and Methods

A website was prepared to conduct this observational prospective cohort study. The website was launched after receiving the approval of the ethics committee in October 2012 (B.30.2.EGE.0.20.05.00/OY/1747-723 decision number: 12-5.2/11) and remained active until June 2015. The investigators received a secure entrance to the website. The electronic case report form for each patient with a thalassemia disease and variant hemoglobins and the signed informed consent form were completed by the investigators. The system was able to detect repeated registries for any patient receiving health care in more than one center. The demographic features and disease characteristics of the patients were reported. Affected births from marriages after 2003 were also investigated and relevant information was collected.

## Results

The overall population with a major hemoglobinopathy comprised 2046 patients from 27 thalassemia centers (TCs) participating in the study. A total of 56 double and one triple registration were excluded. A total of 1988 patients were analysed.

### Distribution of Patients Throughout Turkey

The majority of patients came from TCs in the Aegean (n=622), Marmara (n=518), Mediterranean (n=348), and Southeastern Anatolia (n=338) regions. A total of 139 patients were registered from TCs in Central Anatolia and 23 patients were from a single TC serving the whole of Eastern Anatolia. There was no TC in the Black Sea region where a few patients may be living and receiving health care from the nearest TCs outside the region (Table 1). The highest number of registered patients lived in İstanbul (n=265), İzmir (n=207), and Şanlıurfa (n=201) provinces.

### Demographic Characteristics of Patients

**Figure 1:**
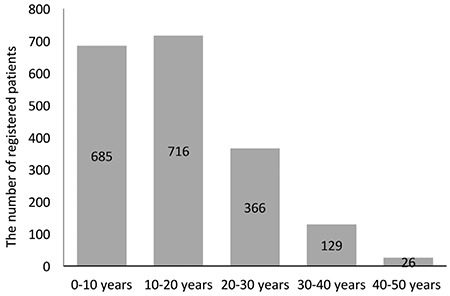
The age distribution of the registered patients.

This was a relatively young cohort (51% male), of which 72% of individuals were below 20 years old (Figure 1). A total of 378 subjects (19%) in the cohort were of preschool age (<6 years). The majority of subjects aged ≥6 years were students (n=981, 67%). A total of 480 subjects (33%) were not attending school. Just over half of these (n=256, 53%) were >18 years old and employed, whereas 224 (47%) were unemployed and 214 of those were >18 years old. Of the unemployed patients 57% had only completed the 8-year primary education, whereas 33% had graduated from high school and 10% from university. The schooling or employment status was not obtained from 149 subjects. All patients, except for 1%, were covered by social security regardless of their social status.

Consanguineous marriage was reported for 48% of parents and 51% of those were first-cousin marriages. Consanguineous marriages accounted for 75% of parents from Şanlıurfa, which was the city with the third highest number of thalassemic patients on the registry. In comparison, consanguineous marriages were reported in 38.5% and 29% of parents from İstanbul and İzmir, respectively. Furthermore, the average number of children born to parents with an affected child was 4 in Şanlıurfa but 2 in İstanbul and İzmir. A total of 214 families in the registry had more than one thalassemic child.

### Disease Characteristics

The majority of subjects (95%) had homozygous b-thalassemia (Table 2). A total of 1385 b-thalassemia alleles reported from 724 patients contained 22 different b-thalassemia mutations. The most common 11 mutations represented 90% of all b-thalassemia alleles. IVS1-110(G->A) was the most prevalent mutation (Table 3).

Although b-thalassemia intermedia (TI) was reported in 215 (11.5%) of 1873 patients with b-thalassemia, only one-third of subjects (33.3%) were entirely transfusion-free. Regular (>8 times/year), frequent (5-8 times/year), and occasional (0-4 times/year) transfusions were reported in 79 (37.6%), 30 (14.3%), and 31 (14.8%) patients, respectively. 

Splenectomy had been performed in 79 (38%) of 207 patients with TI and 590 (37%) of 1594 patients with b-thalassemia major (TM). The patients were divided into four age cohorts by decades and splenectomy indication during the first decade was compared between age cohorts II, III, and IV. The splenectomy frequency in age cohort III displayed a slight decrease compared to cohort IV and simply shifted to the second decade. However, the frequency of splenectomy did not change in age cohort II compared to III (Table 4). 

A total of 115 patients with TM were aged <2 years at the time of registration and had not met the criteria for starting ICT. A total of 150 patients with TI, hemoglobin H (HbH) disease, sickle-cell disease (SCD), and b/S thalassemia were not receiving ICT. The history of ICT was not obtained for 78 patients. Overall, 1561 of 1645 patients (95%) with TM (n=1473), TI (n=128), SCD (n=31), b/S thalassemia (n=9), and HbH disease (n=4) were receiving a monotherapy regimen. DFX was the most prevalent chelator, prescribed to 1337 (81.3%) patients, followed by DFO to 131 (8%) and DFP to 93 (5.7%) patients. Combined therapy of DFO+DFP was reported in 58 (3.5%), DFX+DFO in 20 (1.2%), and DFX+DFP in 6 (0.3%) patients. The highest DFX administration of 93.6% was reported in patients aged <10 years and it remained the most prevalent chelator in all age cohorts. The use of DFO and DFP was lowest in patients aged <10 years and increased gradually in older age cohorts (Table 5).

Hemopoietic stem cell transplantation (HSCT) was reported in 96 patients, of which all but one with SCD had TM. The average age at HSCT was 8.1 years (median: 7 years) and the oldest patient was 18 years old. The source of HSCT was matched sibling donor (MSD) in 87 of 92 patients, whereas three family and two unrelated-donor transplantations were reported. Overall, 70 of 91 patients (77%) had thalassemia-free survival after HSCT, whereas 20 patients had graft rejection with autologous recovery (22%) and 1 died (1.1%). There were 115 patients with an MSD who had not yet had HSCT, of whom 84 were <17 years old. Furthermore, there were 417 patients with a healthy sibling whose human leukocyte antigen (HLA) compatibility had not been evaluated. 

There were 34 deaths (5%) out of 680 patients from 3 TCs. The causes of death were heart disease (n=17), infections (n=8), hepatic failure (n=2), anemia (n=1), HSCT (n=1), and unknown causes (n=5). The earliest cardiac death was at 11 years old. The rates of cardiac deaths in the population at risk (age of >10 years) improved gradually in 5-year cohorts since 1999 (Table 6).

### The Impact of the Hemoglobinopathy Control Program on Thalassemic Births

**Figure 2:**
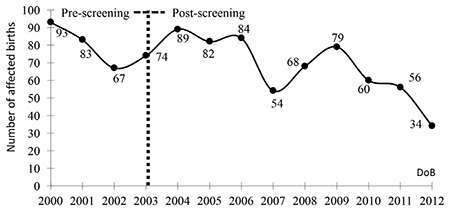
The number of affected births prior to and after the implementation of the hemoglobinopathy control program.

There were 619 thalassemic births after 2004. The number of new cases has shown a consistent decrease only since 2009 (Figure 2). The year of marriage was recorded for 482 of 619 parents, of whom 242 had been married since 2003 or later. According to the statements of couples, overall 142 of those 242 (58.7%) had married in provinces covered by the HCP but did not receive premarital screening. The remaining 100 couples had premarital screening but 40% of those either received no feedback information (n=25) or were misinformed (n=15) regarding screening results and 60% had been informed of being couples at risk of having thalassemic offspring but those parents either had not had a prenatal diagnosis (n=49) or had knowingly given birth to a thalassemic child (n=11). 

Sixty-two of these 242 (25.6%) couples were married in Şanlıurfa. Premarital screening was performed for only 17 (27%) of these 62 couples. Although 12 out of those 17 were informed that they were at-risk couples, only one had a prenatal diagnosis but knowingly gave birth to an affected child. Nineteen (7.8%) of the 242 couples were married in İzmir, of whom 15 (79%) had premarital screening and 10 of those 15 were informed that they were at-risk couples, but only 5 of those had a prenatal diagnosis.

## Discussion

Previous epidemiological studies from Turkey reported that the Çukurova region was the most prevalent for hemoglobin S (HbS) carriers (up to 10%) and the majority of patients with SCD were from that region [4,5,6]. Because the TCs that participated from Çukurova had not registered patients with SCD, the current registry mainly included patients with homozygous b-thalassemia. TI accounted for 11.5% of the cohort and the majority of those individuals were receiving transfusions. It remains to be determined whether the milder forms have been missed. 

Although the prevalence of b-thalassemia carriers was stated as 2.1% overall in Turkey [7], the epidemiological data demonstrated regional differences, with a higher prevalence in coastal areas [5,8,9,10]. In concordance with this, the majority of patients came from the Marmara, Aegean, and Mediterranean regions. Although epidemiological data from Southeastern Anatolia did not indicate a high prevalence of thalassemia carriers [11,12], homozygous forms in the region were found to be as high as those in the coastal areas, most probably because of the higher number of consanguineous marriages and the higher fertility rate. The considerable number of families with more than one affected child indicated that preventive measures have not been implemented even for the families with a proven risk. After implementation of the HCP, the highest number of affected children were born in Şanlıurfa. It was revealed that the majority of these couples had not had premarital screening and, furthermore, prenatal diagnosis was either not offered or not accepted by the at-risk families. The number of newborns with thalassemia and hemoglobinopathies was reported as being reduced from 272 in 2002 to 25 in 2010, which accounted for a 90% reduction over these years [13]. We consider that report with caution since in the current registry 79 affected births were reported from 27 TCs in Turkey in 2010. This inconsistency can be explained by insufficient reporting of new cases to the official registry system used by the Ministry of Health in Turkey. Nevertheless, the number of affected newborns per year demonstrated a trend towards a consistent decrease since 2009. This achievement can be improved by auditing all components of the program carefully and applying appropriate corrective measures. 

This was a relatively young cohort as 72% of the registry was <20 years old and they were mostly either of preschool age (19%) or students (67%). Approximately one-half of the remaining thalassemic subjects were employed while just under half were neither employed nor in education or training (NEET). The Organisation for Economic Co-operation and Development (OECD) reported that nearly 30% of young people in Turkey aged 15-29 were NEET, which is well above the OECD average of 15%, and low skills were a key barrier to achieve better labor market outcomes for youth in Turkey [14]. In fact, 57% of NEET individuals in the registry were early school-leavers. Although the patients were covered by social security regardless of their social status, effective policies are needed to improve the education, job, and career prospects of the patients up to at least the average of their peers. Taking into account that most children and adolescents in this cohort will be moving from childhood to adulthood in the near future, the transition from pediatric to adult care should also be adjusted appropriately.

The wide molecular heterogeneity of Turkish thalassemic subjects has been confirmed by this registry. The most common seven mutations accounted for less than 80% of all thalassemia alleles, consistent with previous reports from Turkey [15,16,17,18,19,20]. The IVS-I-110(G->A) substitution was the most common defect with a frequency of 47% within all b-thalassemia alleles in the cohort. Five of the seven most common b-thalassemia alleles were either b^0 ^(codon 39[C->T], IVSI-1[G->A], FSC8[-AA]) or severe b^+ ^thalassemia (IVSI-110[G->A], IVSII-745[C->G]), whereas only two prevalent alleles (IVSI-6[T->C], IVSII-1[G->T]) were related to mild b^++^-thalassemia mutations. 

It is suggested that improved tissue oxygenation by adequate transfusion regimens has considerably reduced the incidence of splenectomy within the first 10 years of life in thalassemic patients [21,22]. The unchanged needs for splenectomy in our patients from the mid-1970s to mid-2000s may be related to the low transfusion rates in Turkey.

All guidelines provide age-specific recommendations for the initiation of ICT. In children <6 years old, all guidelines recommend DFO as the first-line choice and DFX as the second-line option for patients where DFO is ineffective or not tolerated. DFP is recommended for children >6 years old and/or as a second-line option if patients are resistant or intolerant to DFX [21,23]. Under the regulations of Turkey, all chelators have been approved as first-line treatment at the age of ≥2 years and DFX has been the first-line choice for more than 90% of patients. 

HSCT has remained the only curative treatment for TM. The Turkish Pediatric Bone Marrow Transplantation Group specifically collected the data of 245 thalassemic children who underwent HSCT and of whom 68% achieved thalassemia-free survival [24]. In this registry, only 96 patients were reported as having HSCT. The missing registration data may result from the loss of follow-up of these patients because their health care is usually moved from the TC to the transplantation center after HSCT. Nevertheless, there were 115 TM patients with an MSD but not yet transplanted and a further 417 patients with healthy sibling(s) with unknown HLA compatibility. These data indicate that the awareness of physicians and parents about this curative option should be increased. 

The widespread implementation of cardiac T2* MRI and appropriate intensification of chelation in those with cardiac iron overload reduced cardiac mortality significantly [2,3]. Survival data from three major TCs indicated that despite a gradual improvement in cardiac deaths in the at-risk population in 5-year cohorts since 1994, cardiac disease is still a major cause of early deaths and a sustained effort in dissemination of cardiac T2* MRI and optimum use of ICT should be maintained. The compliance with ICT remained the most important factor in ensuring the desired outcome for thalassemic patients and that may be strengthened by individualized treatment, careful monitoring, and continuous psychosocial support [2,25].

## Conclusion

In conclusion, many efforts have been directed toward optimizing patients’ management and implementing a prevention program in Turkey in the new millennium. The current data indicate that these efforts should be maintained to achieve further improvement in the survival and quality of life associated with better integration into social life for thalassemic patients. The developing strategies are also essential for further achievements in the prevention program.

## Figures and Tables

**Table 1:**
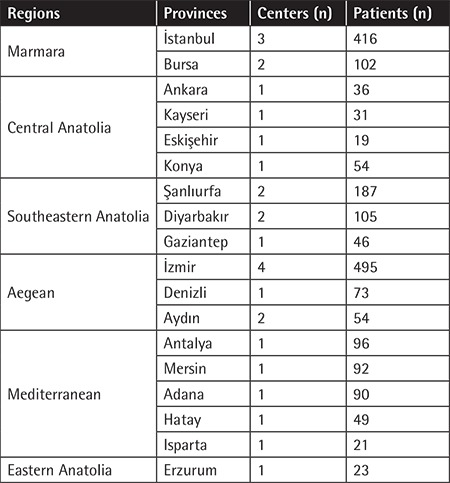
Regional distribution of the registered patients.

**Table 2:**
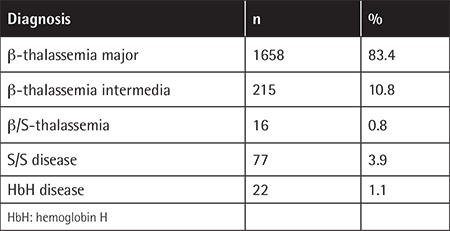
The diagnosis of registered patients.

**Table 3:**
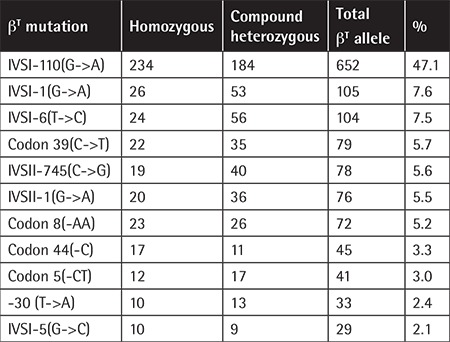
The most common β-thalassemia mutations in the cohort.

**Table 4:**
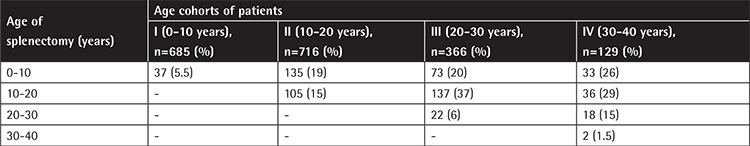
Changes in frequency and age of splenectomy in age cohorts by decades.

**Table 5:**
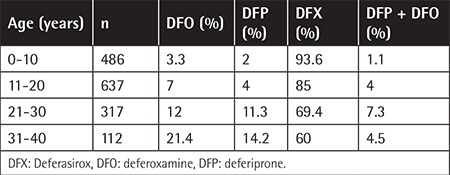
Changes over time in percentage of chelator use in patients with hemoglobinopathies.

**Table 6:**
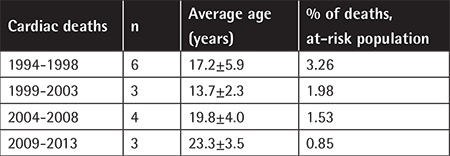
Changes over time in the number and age of cardiac deaths.
